# Extraction of echocardiographic data from the electronic medical record is a rapid and efficient method for study of cardiac structure and function

**DOI:** 10.1186/2043-9113-4-12

**Published:** 2014-09-20

**Authors:** Quinn S Wells, Eric Farber-Eger, Dana C Crawford

**Affiliations:** 1 Department of Medicine, Vanderbilt University, Nashville, TN 37232, USA; 2 Department of Pharmacology, Vanderbilt University, Nashville, TN 37232, USA; 3 Center for Human Genetics Research, Vanderbilt University, Nashville, TN 37232, USA; 4 Molecular Physiology and Biophysics, Vanderbilt University, Nashville, TN 37232, USA; 5 Vanderbilt University Medical Center, 2525 West End Avenue, Suite 300, Nashville TN 37203, USA

**Keywords:** Electronic health records, Echocardiography, Natural language processing

## Abstract

**Background:**

Measures of cardiac structure and function are important human phenotypes that are associated with a range of clinical outcomes. Studying these traits in large populations can be time consuming and costly. Utilizing data from large electronic medical records (EMRs) is one possible solution to this problem. We describe the extraction and filtering of quantitative transthoracic echocardiographic data from the Epidemiologic Architecture for Genes Linked to Environment (EAGLE) study, a large, racially diverse, EMR-based cohort (n = 15,863).

**Results:**

There were 6,076 echocardiography reports for 2,834 unique adult subjects. Missing data were uncommon with over 90% of data points present. Data irregularities are primarily related to inconsistent use of measurement units and transcriptional errors. The reported filtering method requires manual review of very few data points (<1%), and filtered echocardiographic parameters are similar to published data from epidemiologic populations of similar ethnicity. Moreover, the cohort is comparable in size, and in some cases larger than community-based cohorts of similar race/ethnicity.

**Conclusions:**

These results demonstrate that echocardiographic data can be efficiently extracted from EMRs, and suggest that EMR-based cohorts have the potential to make major contributions toward the study of epidemiologic and genotype-phenotype associations for cardiac structure and function in diverse populations.

## Background

Indices of cardiac structure and function are clinically relevant parameters associated with important outcomes. Several measures of cardiac structure, including wall thickness and left ventricular dilation, predict cardiovascular disease events and heart failure [1,2]. Additionally, left atrial size is related to incidence of atrial fibrillation, stroke, and death, and aortic root size is associated with risk of heart failure, stroke, and mortality [1,3,4]. These pathologic changes in cardiac structure and function occur in response to myocardial injury and a wide range of stressful stimuli. Discovery of environmental, physiologic, and genetic factors influencing cardiac remodeling will improve understanding of underlying pathologic mechanisms, and may identify pathways and targets for therapeutic intervention.

Structural cardiac parameters are assessed using a variety of imaging modalities. However, the cost of cardiac imaging can prevent their broad application in large research cohorts. One possible solution to this challenge is to leverage data, obtained during clinical care, found in large EMRs. With the growth of EMRs, there have been considerable efforts to utilize clinical data to support cohort development, clinical research, and genetic research. However, EMR data are complex, often unstructured, and are prone to multiple types of errors and local idiosyncrasies. A key challenge has been the development of strategies to accurately extract high value clinical data and identify phenotypes of interest [5]. Nonetheless, there are multiple examples successful use of EMR-derived data for research, particularly for genetic association studies [1,3,4,6-11].

The most common technique to assess cardiac structure in the clinical setting is transthoracic echocardiography (referred to subsequently as “echocardiography”). These studies routinely acquire important parameters that define cardiac structure including left ventricular septal and posterior wall thicknesses, left ventricular end systolic and diastolic diameters, left atrial diameter, and aortic root diameter. As a potentially important data source in a clinically relevant population, echocardiographic information within EMRs is subject to multiple sources of error, including incorrect data entry, transcriptional errors, and inconsistent use of measurement units. Moreover, the data may not be structured in such a way that extraction is straightforward. As such, rapid and efficient methods for the extraction and filtering of echocardiographic data from EMRs are needed. In this report, as part of the Epidemiologic Architecture for Genes Linked to Environment (EAGLE) study, we describe the extraction and filtering of six canonical quantitative echocardiographic variables including left ventricular septal thickness, left ventricular posterior wall thicknesses, left ventricular end systolic diameter, left ventricular end diastolic diameter, left atrial diameter, and aortic root diameter in an racially diverse population from a large EMR for eventual genetic association studies.

## Methods

### Study population

The Vanderbilt University Medical Center (VUMC) biorepository, BioVU, is a resource linking DNA samples to a de-identified EMR, termed the Synthetic Derivative (SD), that contains approximately 20 years of data on over 2 million individuals. The SD is generated by the application of a one-way hash to the EMRs that removes or de-identifies protected health information such as proper names, geographical locations, medical record numbers, and social security numbers. Dates are randomly shifted by up to six months (but consistently within any single record). As of March 2014 BioVU houses DNA samples from over 175,000 subjects. The design and implementation of the Synthetic Derivative and BioVU have been previously described, [12] as has utilization of the resource for replication of known associations between genetic variants and common diseases [13].

EAGLE, as part of the larger Population Architecture using Genomics and Epidemiology I (PAGE I) study, [14] selected 15,863 BioVU samples (EAGLE BioVU) from diverse populations for genotype-phenotype studies, including 11,503 African Americans, 1,702 Hispanics, and 1,098 Asians, to generate a near-complete cross-section of all minority populations in BioVU as of 2012. Subjects have been phenotyped for a range of important characteristics including body mass index (BMI), serum lipid levels, renal function, and hemoglobin A1C Dumitrescu L, Goodloe R, Boston J, Farber Eger E, Pendergrass SA, Bush WS, Crawford DC: Towards a phenome-wide catalog of human clinical traits impacted by genetic ancestry, submitted]. We restricted echocardiographic data extraction and analyses to the 13,957 non-white adult (age ≥ 18 years) subjects within EAGLE BioVU.

### Extraction of echocardiographic data from EMRs

Echocardiography reports in the VUMC EMR are in the portable document format (PDF) and have undergone three formatting iterations since 1997. Reports prior to 1997 are not in digital format and not included in the EMRs. Each report contains structured, semi-structured, and unstructured data. Structured data are generally quantitative measures such as wall thicknesses, chamber dimensions, or flow velocities. Semi-structured data fields contain subjective interpretations of parameters with a limited number of potential values. These fields frequently contain ordinal data. For example, valvular lesions and abnormalities of ventricular function are often subjectively quantified as “mild”, “moderate”, or “severe”. Unstructured fields contain unrestricted prose descriptions of clinically relevant findings as interpreted by the reader.

Fields containing structured, semi-structured, and unstructured data were identified within echocardiography reports in the EMRs. Numeric values for left ventricular septal thickness, left ventricular posterior wall thicknesses, left ventricular end systolic diameter, left ventricular end diastolic diameter, left atrial diameter, and aortic root diameter were subsequently parsed from reports using natural language processing.

### Systematic filtering of quantitative echocardiographic data

#### Step 1: Identification and characterization of outliers

We first examined the distributions of and relationships between quantitative echocardiographic parameters. Extreme outliers, unrealistic values, and unusual relationships (i.e., relative values between parameters) were identified and evaluated manually, including comparison with other echocardiogram reports. Data points were retained when found to be valid, edited when obvious data entry errors were identified, and removed in all other cases.

**Figure 1:**
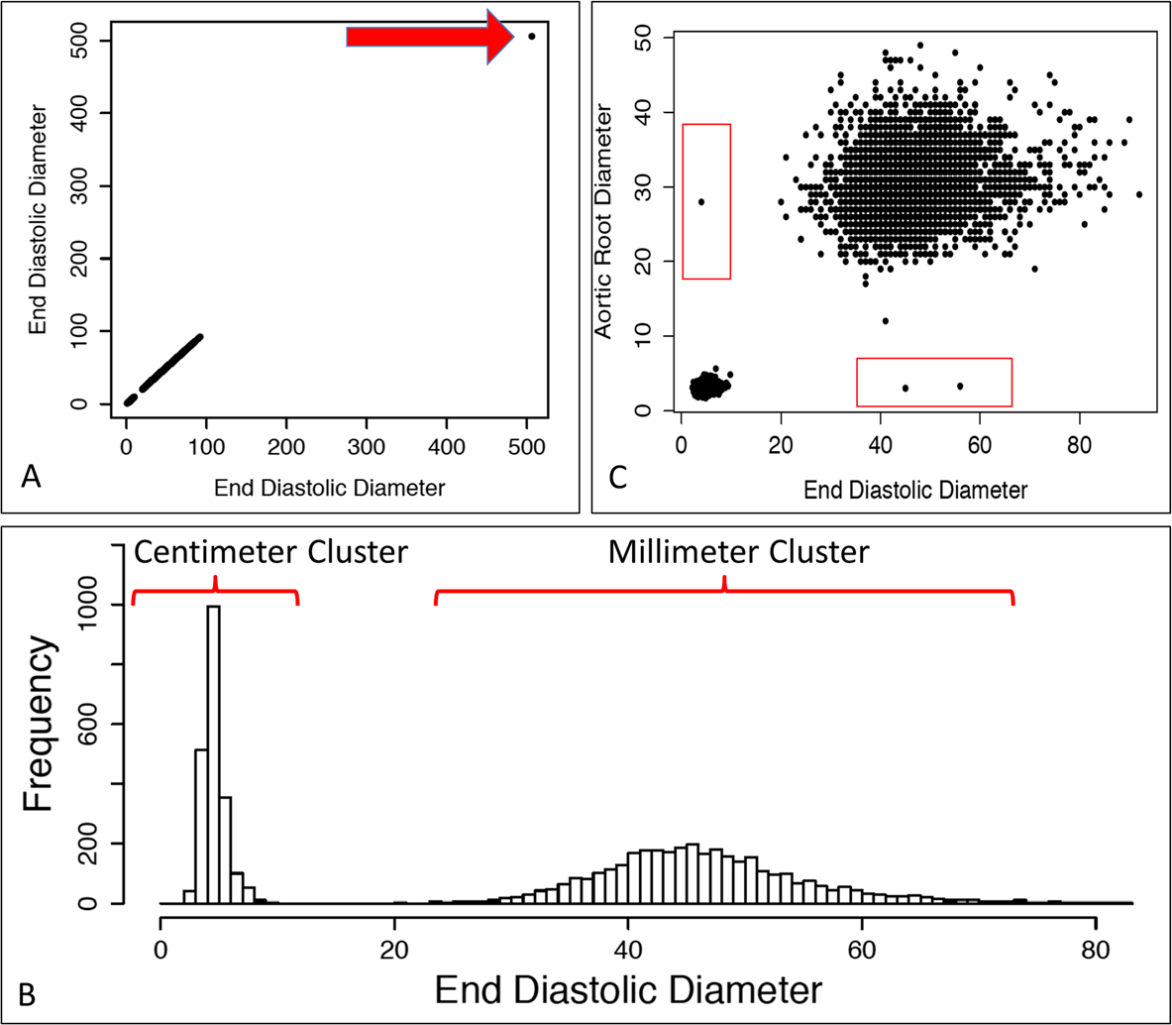
**Representative graphical representations of raw quantitative echocardiographic parameters. (A)** Identity plot of end diastolic diameters highlighting extreme outlier (arrow). **(B)** Histogram of end diastolic diameter showing two distributions related to measurement units. **(C)** Representative pairwise scatterplot (in this case end diastolic diameter vs aortic root diameter) shows two primary clusters related to measurement units, but also outliers with discordant units (red boxes).

Examination of raw data revealed both systematic and non-systematic data inconsistencies. Non-systematic irregularities generally arose due to transcriptional errors (e.g., input of nonsensical values or entry of measurements into inappropriate fields), while systematic discrepancies were related to use of different measurement units – specifically centimeters versus millimeters. For most reports, the use of one unit or the other was consistent and dictated by institutional practices at the time the echocardiogram was performed. However, some reports were found to have internally inconsistent use of measurement units.Figure 1 shows representative graphical displays used to identify error sources in raw data. Panel A demonstrates how identity plots for each parameter were used to detect extreme outliers and data points with unrealistic values. In this example, a single extreme outlier is identified in left ventricular end diastolic diameter. In total, there were 16 extreme outliers from the six parameters identified for manual review. Two were clearly due to transcriptional errors (e.g., systolic diameter entered into diastolic diameter data field and vice versa). In these cases, data were manually edited and retained. Three values (2 left atrial diameter and 1 septal thickness) were confirmed to be accurate. In all other cases values were removed. After removal of extreme outliers, histograms of each parameter, as in Panel B (left ventricular end diastolic diameter again shown as an example), demonstrate distinct distributions due to use of different units. Finally, pairwise scatterplot of all parameters, as shown in Panel C (scatter plot of left ventricular end diastolic diameter versus aortic root diameter is shown as an example), confirm the presence of two distributions related to units, and also reveal another distinct class of outlier related to inconsistent use of units within individual reports.

#### Step 2: Identification of measurement unit-discordant outliers

**Figure 2:**
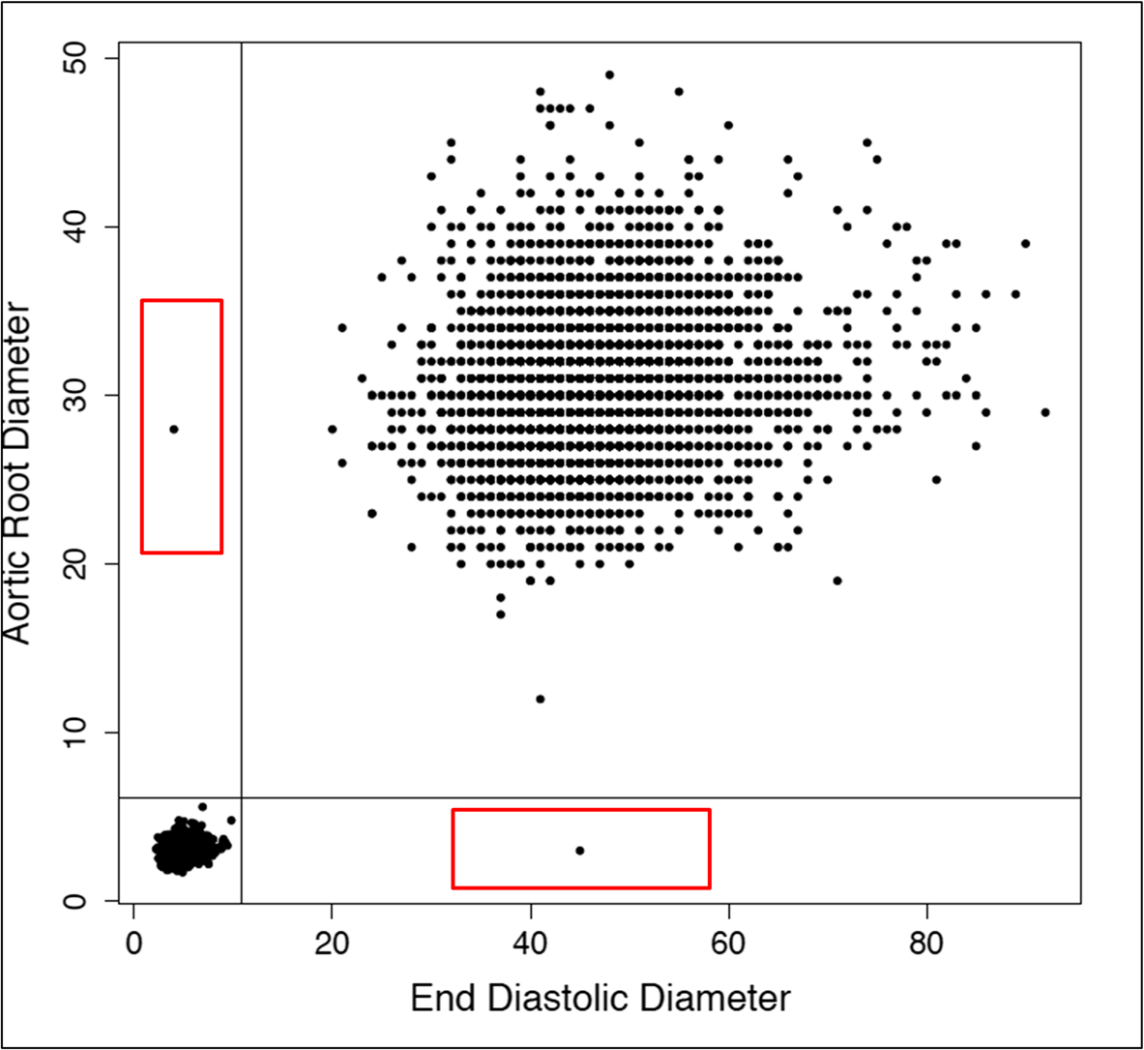
Representative graphical representation of data points in reports with inconsistent use of measurement units (red boxes).

After removal of extreme and unrealistic values, data were next evaluated for unusual parameter relationships characteristic of unit-discordant data points. The procedure began with examination of scatterplots for all pairwise combinations of parameters. For each scatterplot, maximum values for each parameter were extracted from the cluster closest to the origin, and 110% of this value was used to define thresholds for each parameter below which data points could reliably be assumed to be in centimeters. For instance, in Figure 2, where a scatter plot of left ventricular end diastolic diameter versus aortic root diameter is shown, the maximum value for left ventricular end diastolic diameter and aortic root diameter are 9.8 and 5.6, and therefore the lines of 110% of these maximum values are 10.78 and 6.16 respectively. Plotting these thresholds on the scatterplots defines sectors with unit-discordant data points. Unit-discordant data points were converted from millimeters to centimeters (i.e., dividing the discordant parameter value by 10). A total of 55 unit-discordant data points were identified (red boxes) and converted using the procedure described above.

#### Step 3: Check of anatomically constrained relationships

**Figure 3:**
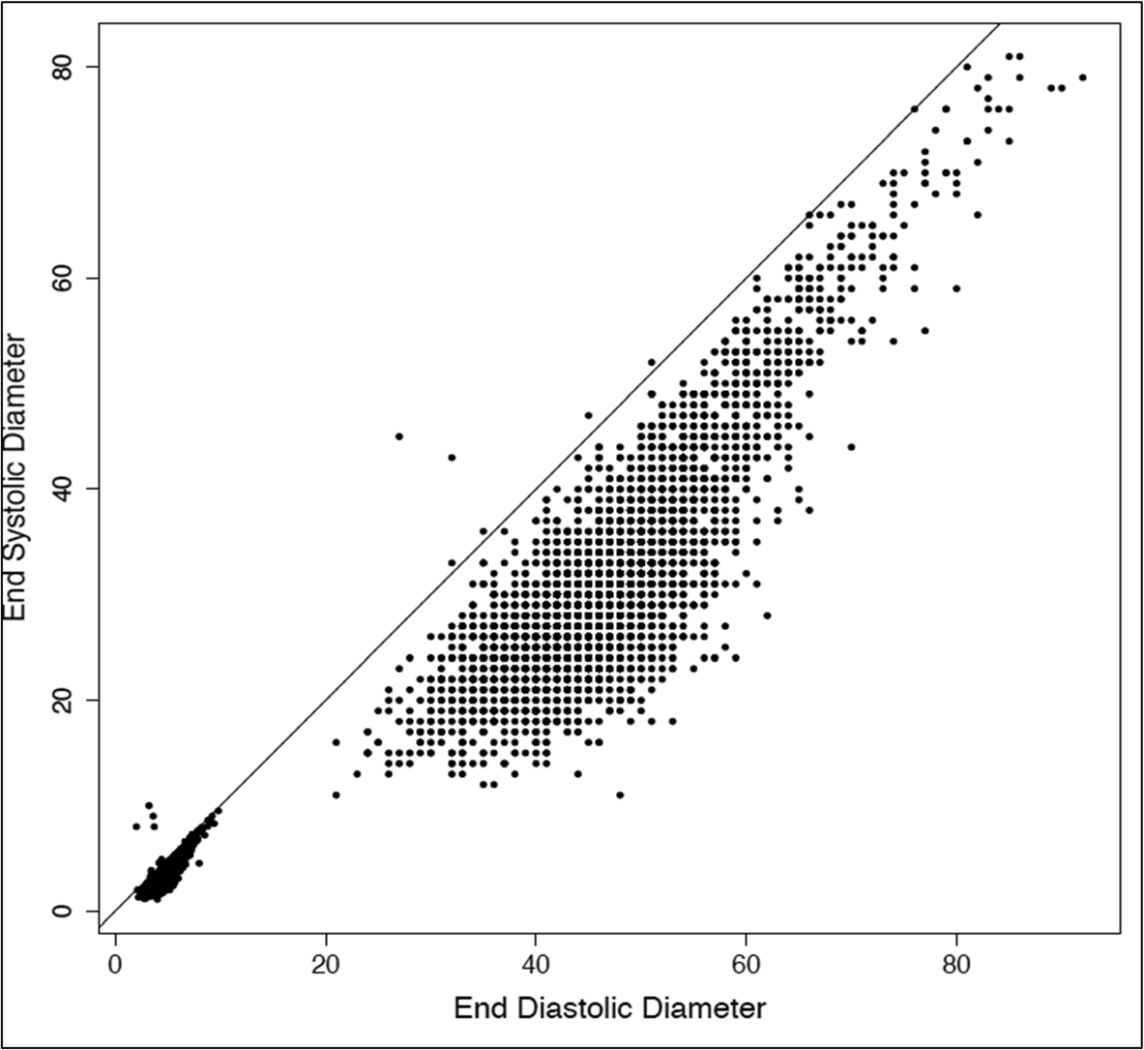
**End systolic diameter plotted against end diastolic diameter.** Data points above the line have anatomically impossible diastolic to systolic diameter ratios.

One characteristic of echocardiographic data that may be leveraged for data filtering is the relationship between systolic and diastolic diameters. Of necessity, systolic measures must be smaller than diastolic values. Thus, records with systolic diameter to diastolic diameter ratios ≥ 1 are anatomically impossible and were identified for manual review. This is shown in Figure 3 where all points above the diagonal line are records containing anatomically impossible values. Manual review was performed on 16 records (32 data points) found to have systolic diameter to diastolic diameter ratios ≥ 1. For 3 records, systolic and diastolic diameters measurements were clearly entered into opposite data fields. In these cases, values were switched and retained. Two records contained systolic diameter values that were found to be aberrant and removed. For the remaining 11 records the source of error was unclear and both values were removed.

#### Step 4: Harmonization of measurement units

**Figure 4:**
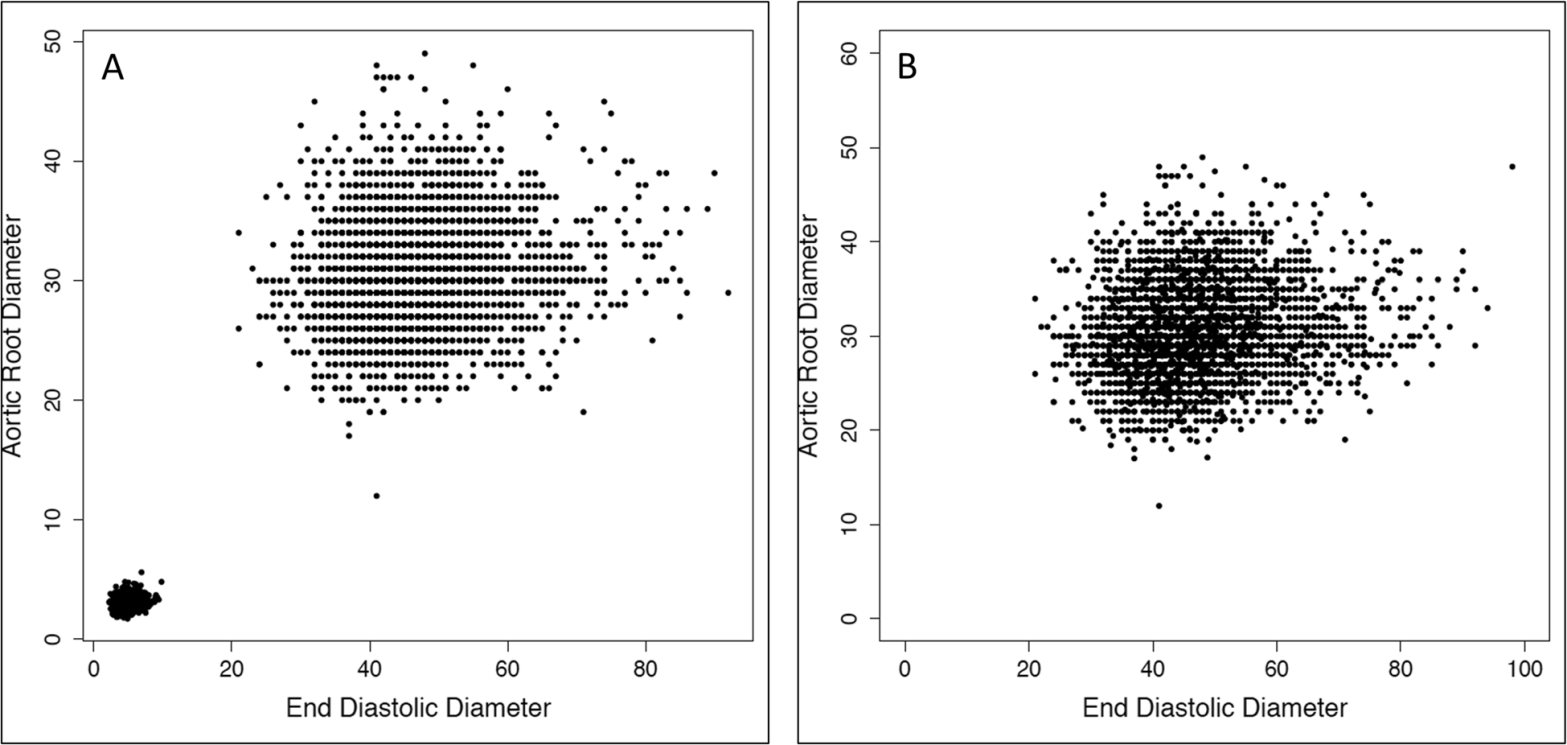
**Data before (Panel A) and after (Panel B) harmonization of measurement units.** Records using centimeters have been converted to millimeters.

After filtering of outliers, unit-discordant records, and records containing anatomically impossible values, remaining data points clustered into two distributions reflecting use of different measurement units. Reports using centimeters were extracted using the thresholds for each parameter defined in Step 2 and converted to millimeters by applying a factor of 10. There were 2,132 reports utilizing centimeters for measurements units. After unit conversion, no new extreme outliers were identified (as would be expected with the inadvertent application of a factor of 10 to an occult value in millimeters), suggesting that our procedure successfully identified reports consistently using centimeters. Figure 4 shows a representative scatterplot before and after unit conversion.

#### Step 5: Final review for residual aberrant data points

Data were again displayed graphically, as in the initial characterization of erroneous data, to identify residual outliers. There were 10 residual outliers identified for review after re-examination of the data (2 aortic root, 3 posterior wall thickness, 5 septal thickness). Six values were confirmed to be accurate (1 aortic root, 1 posterior wall thickness, 4 septal thickness) and retained while 3 (2 posterior wall thickness, 1 septal thickness) were aberrant and removed from the dataset. One aortic root outlier proved to be entered into an incorrect data field (swapped with atrial diameter) and was retained after editing.

### Evaluation of the filtering strategy

The cleaning process was semi-automated in that suspect data points identified at each step underwent manual review to determine their accuracy. Remaining data points were assumed to be free of the specified error types and handled automatically. The accuracy of the filtering strategy for error identification was determined by calculating the proportion data points selected for review that were, in fact, erroneous as determined by manual cardiologist review. The presence and frequency of error types not included in the filtering strategy, missed errors, and introduced errors was determined by comparing the filtered values from 500 random reports to a gold standard generated manually by a cardiologist.

## Results and discussion

**Figure 5:**
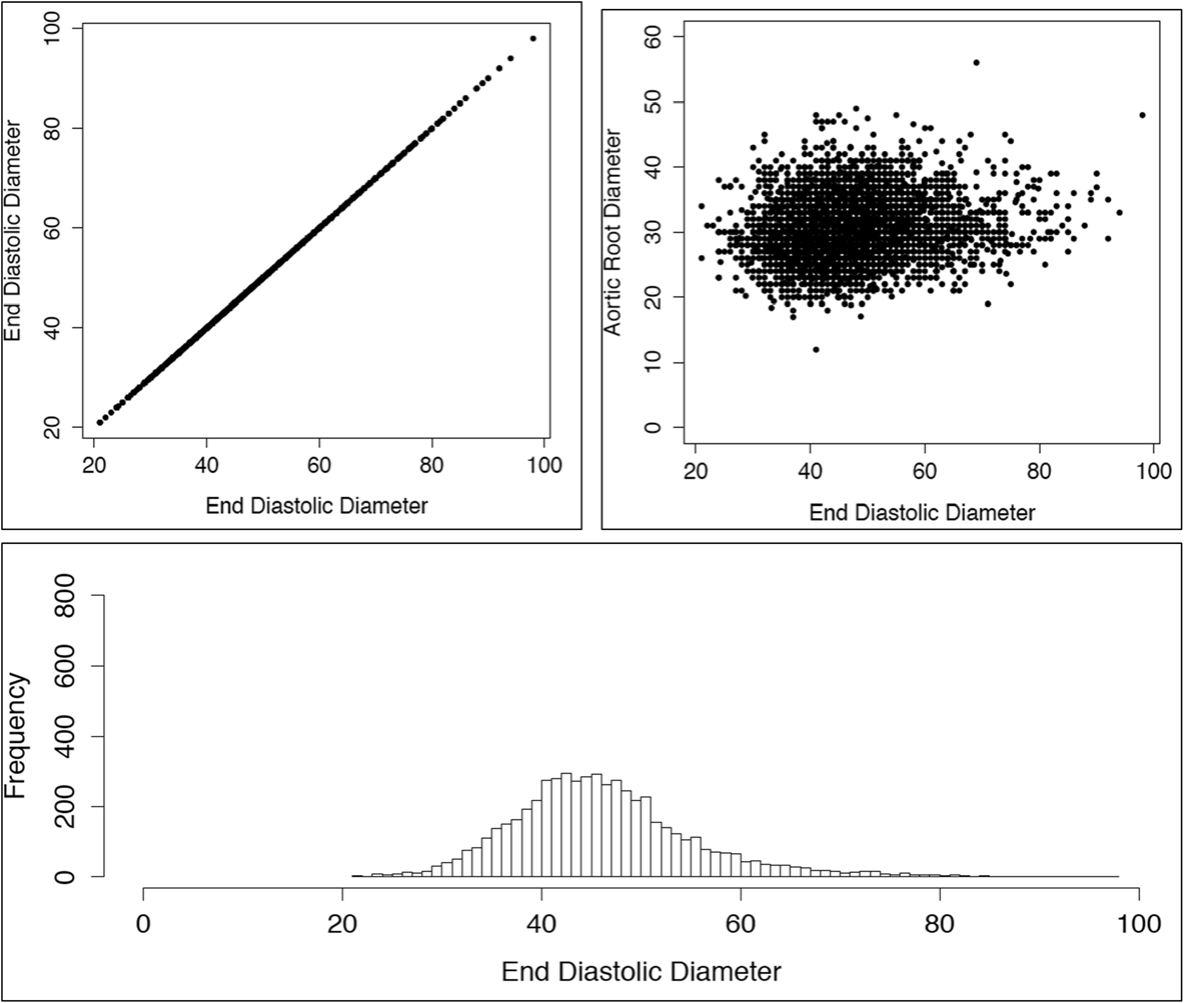
Representative graphical representations of cleaned data.

A total of 6,076 echocardiography reports were extracted from the EAGLE BioVU cohort. Six quantitative structural parameters were targeted, thus 36,456 potential data points (6,076 × 6) were in the complete dataset. Missing data were uncommon with 33,177 values (91%) present. Examination of the distributions of and relationships between echocardiographic parameters after filtering showed the data to be of one distribution and free of extreme outliers (Figure 5).

During filtering, manual review was performed on 113 data points (16 extreme outliers from Step 1; 55 unit-discordant data points from Step 2; 32 anatomically impossible values from Step 3; 10 residual outliers from Step 5), representing less than 1% of the dataset, and no reports were lost entirely. Among manually reviewed data points, only 11 were determined not to contain errors. Thus, 102 of 113 data points were correctly identified, yielding an accuracy of 90% for error identification. Using expert manual review, errors for 64 reviewed data points were successfully cleaned while 38 were removed from the dataset due to unresolvable errors. Among the 500 reports randomly selected to evaluate the performance of the filtering strategy there were 2,612 data points. Expert manual review found no errors that evaded cleaning (sensitivity to error detection ~100%), and there was perfect agreement between the cardiologist and the output of the filtering method.

The filtering method was highly accurate in regard to selection of data points for review, and also produced high sensitivity in regard error detection and agreement with manually curated gold standard. While data regarding the extraction and filtering of quantitative structural data from clinical echocardiography reports is limited, the performance of the current approach was similar reported for extraction of ejection fraction and semi-structured echocardiographic data. For example, one report demonstrated that reduced cardiac function (defined as ejection fraction <40%) can be identified within echocardiography reports with a sensitivity of 98.4% and specificity of 100%, [15] and others have extracted semi-structured data elements with a sensitivity and specificity of 78% and 99% respectively [16].

**Figure 6:**
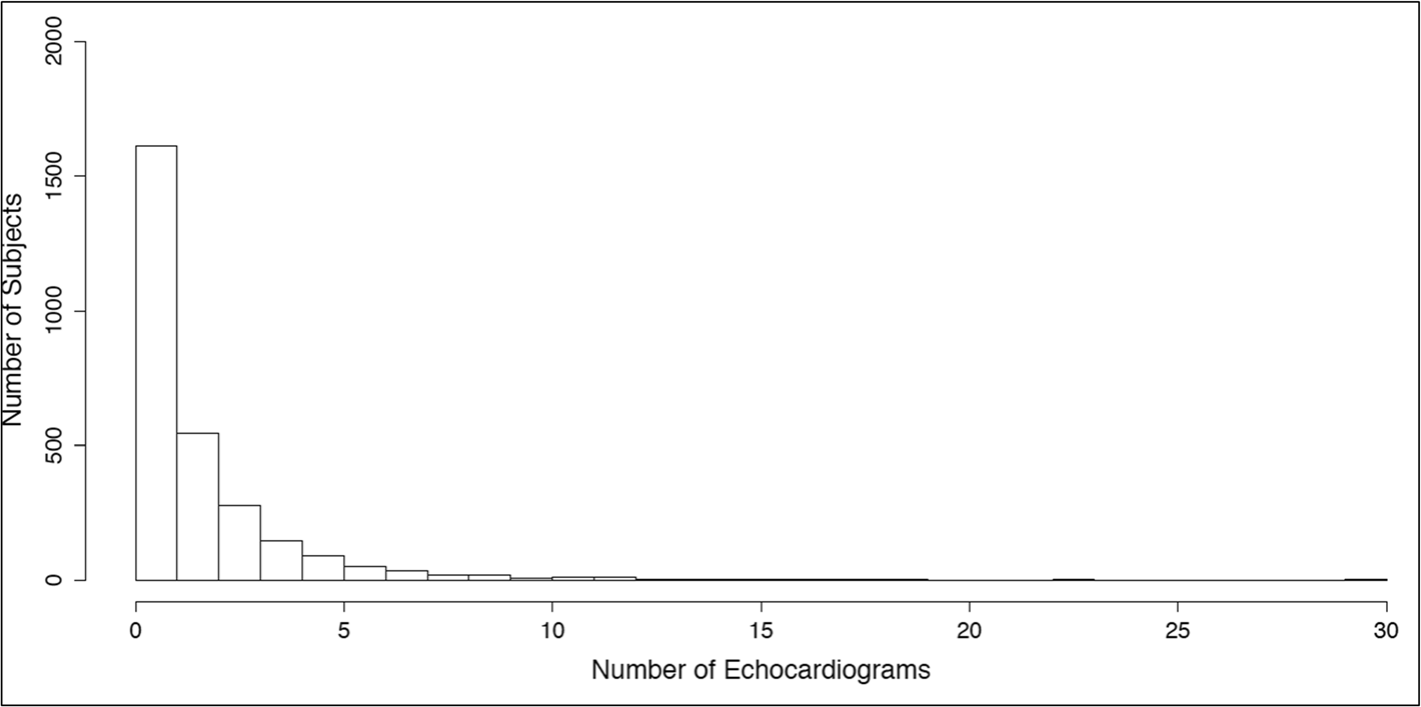
Frequency of echocardiograms for those subjects with at least 1 echocardiogram performed.

The 6,076 extracted reports were distributed among 2,834 unique adult subjects. Thus, 20.3% of the adult EAGLE BioVU cohort had at least 1 echocardiogram. The median number of echocardiograms per subject was 1 [interquartile range or IQR 1–2], with a range of 1 to 30 (Table 1). Figure 6 shows the frequency of echocardiograms per subject for those with at least one procedure. Adult subjects in EAGLE BioVU are, on average, middle aged, majority female, and primarily African American (Table 2). There were significant differences between subjects who did and did not undergo echocardiography. Those with echocardiograms were more likely to be male in addition to being significantly older, and having higher BMI, hemoglobin A1C, and total cholesterol values (Table 3). Published echocardiographic data in non-European descent populations is limited. However a recent genome-wide association study (GWAS) meta-analysis of echocardiographic traits in African Americans reported data from the African American subsets of several community-based cohorts including the Coronary Artery Risk Development in Young Adults Study (CARDIA), the Atherosclerosis Risk in Communities (ARIC) cohort, and the Jackson Heart Study (JHS) [17]. The EAGLE BioVU echocardiography cohort, although clinic-based, is similar to these community-based cohorts in gender distribution and age (aside from CARDIA, which specifically ascertained a younger population) as well as values for echocardiographic parameters (Table 4). Interestingly, the size of the EAGLE BioVU echocardiography cohort is larger than ARIC and CARDIA and comparable to the JHS.

**Table 1:** Echocardiograms in EAGLE BioVU

Total subjects	15,863
Adults	13,958
Unique Echocardiograms	6,076
Unique adult subjects with an echocardiogram	2,834
Median [IQR] echocardiograms/subjects	1 [1, 2]
Range of echocardiograms/subjects	1–30

Reported values represent number of subjects, median [IQR], or range as appropriate.

**Table 2:** Demographics of entire adult cohort in EAGLE BioVU (N = 13,957)

Age (years)	47.5 [33.1–61.1]
Gender (female)	65.4%
Race/Ethnicity	
Black	74.4%
Hispanic	9.0%
Asian	7.0%
Other	9.0%
Body mass index (kg/m^2^)	28.5 [24.1–33.8]
HbA1C (%)	6.1 [5.5–7.0]
Serum creatinine (mg/dL)	0.87 [0.71–1.1]
Total cholesterol (mg/dL)	182 [157–209]
LDL (mg/dL)	105 [83–129]

Values are reported as median [IQR] or percent (%) as appropriate.

**Table 3:** Demographics among individuals with and without echocardiography performed

**Echocardiography performed**				**Echocardiography not performed**				**P = X**^**2 **^**or t-test**
N	2,834 (20.3%)			N	11,123 (79.7%)			<2.2×10^-16^
Age (years)	58.6 [46.2–70.2]			Age (years)	43.8 [31.3–58.1]			<2.2×10^-16^
Gender (female)	1,694 (59.8%)			Gender (female)	7,429 (66.8%)			2.9×10^-12^
Race	N	Within group (%)*	Within race (%)**	Race		Within group (%)*	Within race (%)**	
Black	2,426	85.6	23.4	Black	7,959	71.6	76.6	<2.2×10^-16^
Hispanic	119	4.2	9.5	Hispanic	1,130	10.2	90.5	
Asian	121	4.3	12.2	Asian	869	7.8	87.8	
Other	168	5.9	NA	Other	1,165	10.4	NA	
BMI (kg/m^2^)	29.6 [24.8–35.3]			BMI (kg/m^2^)	28.2 [23.9–33.3]			4.1×10^-16^
HbA1C (%)	6.2 [5.7–7.3]			HbA1C (%)	6.0 [5.4–6.9]			3.7×10^-7^
Serum creatinine (mg/dL)	1.0 [0.8–1.5]			Serum creatinine (mg/dL)	0.8 [0.7–1.03]			<2.2×10^-16^
Total cholesterol (mg/dL)	184 [158–213]			Total cholesterol (mg/dL)	181 [157–208]			0.02
LDL (mg/dL)	107 [84–132]			LDL (mg/dL)	105 [83–128]			0.06

Values are reported as median [IQR] or percent (%) as appropriate.

*Percentage of subjects with or without an echocardiogram performed respectively.

**Percentage of subjects within the specified race.

**Table 4:** Demographic and echocardiographic data for African American subjects from population-based cohorts in published GWAS for echocardiographic traits

	**EAGLE BioVU**			**Atherosclerosis risk in communities**			**Coronary artery risk development in young adults study**			**Jackson heart study**		
	**Women**	**Men**		**Women**	**Men**		**Women**	**Men**		**Women**	**Men**	
Age (mean ± SD)	57.9 ± 17.6	58.6 ± 15.9	P = 0.27	59 ± 6	59 ± 6	P = 1	30 ± 4	29 ± 4	P = 3.4x10^-6^	55 ± 13	54 ± 13	P = 0.04
Echocardiographic traits												
N	1,694	1,140	P-value	698	415	P-value	854	589	P-value	1,884	1,115	P-value
LV diastolic dimension, mm	44.4 ± 7.6	48.5 ± 8.8	<2.2×10^-16^	46 ± 6	49 ± 6	<2.2×10^-15^	48 ± 4.5	51 ± 4.7	<2.2×10^-16^	49 ± 4.1	51 ± 4	<2.2×10^-16^
Left atrial dimension, mm	37.4 ± 7.3	40.1 ± 8.0	<2.2×10^-16^	39 ± 6	39 ± 6	1	35 ± 5	36 ± 4.8	1.4×10^-4^	Not Reported	Not Reported	NA
Aortic root diameter, mm	28.4 ± 3.6	32.8 ± 4.1	<2.2×10^-16^	30 ± 4	34 ± 4	<2.2×10^-16^	26 ± 3	30 ± 3.5	<2.2×10^-16^	30 ± 2.8	34 ± 3	<2.2×10^-16^
Posterior wall thickness, mm	10.5 ± 2.2	11.4 ± 2.5	<2.2×10^-16^	11 ± 2	12 ± 2	<2.4×10^-15^	8 ± 1	9 ± 1.4	<2.2×10^-16^	8 ± 1	9 ± 1	<2.2×10^-16^
LV systolic dimension, mm	29.0 ± 8.4	33.3 ± 10.7	<2.2×10^-16^	Not Reported	Not Reported	NA	Not Reported	Not Reported	NA	29 ± 4	32 ± 5	<2.2×10^-16^
Interventricular septal wall thickness, mm	11.0 ± 2.5	12.0 ± 2.8	<2.2×10^-16^	12 ± 2	12 ± 3	1	9 ± 2	10 ± 1.6	<2.2×10^-16^	9 ± 1	9 ± 1.5	1

Values are expressed in mean ± standard deviation or number of subjects as appropriate.

Adapted from reference [17].

We extracted echocardiographic traits recorded in the EMRs for 2,834 individuals in EAGLE BioVU, the majority of which are African American (85.6%). Our experience shows that extraction and filtering of these parameters can be done rapidly and efficiently. Missing data are uncommon, with only ~10% of data points being absent. There are systematic errors mostly related to choice of measurement units that are easily corrected. However, there are also non-systematic outliers due to inconsistent use of units and transcriptional errors. Nonetheless, our method required manual review of very few data points (<1%). So, while we chose to have these values reviewed by a content expert and retained as possible, there would be a low penalty, in terms of data loss, for simply removing them.

Several important features of the EAGLE BioVU echocardiography cohort are worthy of note. First is its relatively large size, which is similar to, or larger than, several population-based cohorts of African Americans used in previous genetic studies of echocardiographic traits. In addition, the EAGLE BioVU echocardiography population also has demographic and echocardiographic characteristics similar to those of African American subjects from community-based cohorts. Given the difficulties and cost associated with the ascertainment and phenotyping of large cohorts, clinic-based populations, such as the one developed here, represent an important complementary methodology.

There are limitations to the data extraction and cohort development methods presented here. First, of necessity, the EAGLE BioVU echocardiography population is limited to individuals for whom clinically indicated echocardiography was performed at a single, tertiary care, referral center. Additionally, echocardiographic measures were obtained under clinical standards of care and not using standardized research protocols. As such, the EAGLE BioVU echocardiography cohort is subject to the ascertainment biases and data heterogeneity concerns inherent to all clinic-based populations. Finally, only structured, quantitative echocardiographic measures of cardiac structure were extracted. There are clinically important semi-structured and unstructured data, including parameters of myocardial contractility, valvular function, and recognized patterns of disease that were not extracted.

The decision to focus initially on quantitative data was made because these data are critical for the study of cardiac structure (i.e., they define cardiac structure), are often recorded in tabular format, making them more amenable to extraction, and are particularly susceptible to transcriptional errors and measurement unit inconsistencies that must be removed prior to analysis. Semi-structured data, in general, consist of simplified vocabulary explicitly stating the presence or absence of a limited range of conditions (e.g., valvular stenosis or insufficiency), and, if present, the degree of disease (e.g., mild, moderate, severe). Information extraction methods exploiting these characteristics to identify canonical concepts and corresponding values have been described, [16] but these approaches are less robust for rare phenomena and atypical expressions common in large clinical datasets. Expanding the concept-value vocabulary of such tools could mitigate these limitations, but manual review may always be required to filter unusual findings and phrasings. In general, filtering of semi-quantitative echocardiographic data is less of a concern because, possible values for each variable are more constrained (e.g., absent, mild, moderate, severe), limiting the problem of extreme outliers due to transcriptional errors, and values have no units, obviating the need to assure unit harmonization.

Despite these limitations, this report demonstrates that quantitative echocardiographic data can be extracted quite efficiently from large EMRs. Moreover, the comparable size, demographics, and echocardiographic measures of this population to epidemiologic cohorts provide reassurances that, despite many intrinsic biases, EMR-derived datasets are useful for the study of cardiac structure and function.

## Conclusion

These results demonstrate that extraction of echocardiographic data from the EMR environment can be rapid and efficient, and suggest that EMR-based cohorts have the potential to be important data sources for the study of cardiac structure and function. Future directions include refinement of methods for extraction and filtering of semi-structured and unstructured echocardiographic date from EMRs and leveraging of the EAGLE BioVU echocardiography cohort for study of cardiac structure and function genotype-phenotype associations.

## Abbreviations

EMR: Electronic medical records; EAGLE: Epidemiologic Architecture for Genes Linked to Environment study; VUMC: Vanderbilt University Medical Center; SD: Synthetic Derivative, a de-identified version of the VUMC EMR used for research; PDF: Portable document format; GWAS: Genome-wide association study; CARDIA: Coronary Artery Risk Development in Young Adults Study; ARIC: Atherosclerosis Risk in Communities cohort; JHS: Jackson Heart Study.

## Competing interests

The authors declare that they have no competing interests.

## Authors’ contributions

QSW contributed to study design, study execution, data analysis/interpretation, and manuscript preparation. DCC contributed to study design, study execution, data analysis/interpretation, and manuscript preparation. EFE contributed to study execution and manuscript preparation. All authors read and approved the final manuscript.
